# Incidence of intra-abdominal hypertension after liver transplantation with temporary portocaval shunt – A prospective cohort

**DOI:** 10.1016/j.clinsp.2026.100900

**Published:** 2026-03-11

**Authors:** Estrella Bianca Melo, Luiz Augusto Carneiro-D’Albuquerque, Wellington Andraus, Rodolpho Augusto Pedro, Bruna Carla Scharank, Paula Sepulveda Mesquita, Daniel Reis Waisberg, Rodrigo Bronze de Martino, Vinicius Rocha-Santos, Liliana Ducatti, Lucia C. Andrade, Alberto Queiroz Farias, Luiz Marcelo Sá Malbouisson

**Affiliations:** Hospital das Clínicas da Faculdade de Medicina da Universidade de São Paulo, São Paulo, SP, Brazil

**Keywords:** Abdominal compartment syndrome, Intra-abdominal hypertension, Liver transplantation

## Abstract

**Background:**

Intra-Abdominal Hypertension (IAH) is reported to be frequent after liver transplantation. Nonetheless, the evolution of surgical techniques improved postoperative care in the last decades, which could lead to a lower incidence of Intra-abdominal hypertension.

**Methods:**

The authors prospectively studied 104 patients. The intra-abdominal pressure and abdominal perfusion pressure were measured at the end of surgery and every 6 hours until 72 hours, and patients were divided into two groups: with (IAH^+^) or without (IAH^-^) IAH. Outcomes analyzed were death, renal function, reoperation, need for vasopressors, mechanical ventilation, hemodialysis, and graft function measured by indocyanine green.

**Results:**

The incidence of Intra-abdominal hypertension was 14.4%, with no abdominal compartment syndrome cases. After 48 hours, 60% of the IAH^+^ group was on mechanical ventilation versus 26.7% in the IAH^-^ group (*p* = 0.016). The prevalence of dialysis at the end of the first postoperative week was higher in the IAH^+^ group (31.3% vs. 10.8%, *p* = 0.047). Both groups had similar indocyanine green clearance on the third and seventh postoperative days. Reoperation rates (12.4% vs. 20%), need for retransplant (10.1% vs. 13.3%), and death (14% vs. 18.2%) were similar in IAH^-^ and IAH^+^ groups, respectively. The number of blood products transfused (OR = 1.18) and body mass index (OR = 1.10) were associated with the development of intra-abdominal hypertension, while intraoperative ascites (OR = 0.19) was a protective factor.

**Conclusions:**

Intra-abdominal hypertension was present in 14.4% of liver transplant patients, and IAH was linked to a greater need for mechanical ventilation and hemodialysis but not to more reoperations, transplants, or deaths.

## Highlights

• The incidence of intrabdominal hypertension was low in this study.
• IAH was linked to a greater need for mechanical ventilation.
• IAH was linked to a greater need for hemodialysis.
• No cases of abdominal compartment syndrome were observed.

## Introduction

Intra-Abdominal Hypertension (IAH) is linked to postoperative organic dysfunction, increased intensive care length of stay, and high mortality rates^1^ This issue is especially relevant in Orthotopic Liver Transplant (OLT) recipients, in whom the incidence of IAH and Abdominal Compartmental Syndrome (ACS) has been reported to be as high as 48 % and 15 %, respectively^2^, ^3^, ^4^ Intra-abdominal hypertension is associated with large liver grafts, multiple transfusions, and bowel edema secondary to intensive fluid resuscitation and vascular clamping^3^, ^4^, ^5^ Acute Kidney Injury (AKI) and extended mechanical ventilation after OLT have been reported in up to 25 % of patients with IAH, with a 50 % mortality rate when renal replacement treatment is necessary^1^

Fortunately, in the last two decades, a greater understanding of intra-abdominal physiology, more precise definitions of IAH/ACS^6^^,^^7^ and improved perioperative care have led to a more efficient management of IAH complications^8^, ^9^, ^10^, ^11^, ^12^ Some strategies, such as temporary portocaval shunt, restrictive fluid management, and thromboelastography-guided transfusion, routinely used in this service, could further decrease the incidence of IAH after OLT. However, this data is rarely described in the latest literature. Therefore, the primary objective of this prospective cohort is to describe the incidence, risk factors, and clinical impact of IAH following OLT with a temporary portocaval shunt.

## Methods

This cohort study prospectively included 104 patients undergoing Brain-Dead Donor Liver Transplantation (BDDLT) at the *Hospital das Clínicas da Faculdade de Medicina da Universidade de São Paulo* from September 2017 to September 2019. The study was temporarily halted from July 2018 to March 2019 due to a worldwide recall of the AbViser® system by its manufacturer, ConVaTec^13^ After this period, the recruitment resumed according to schedule and continued uninterrupted until the study's conclusion.

The study was approved by the institutional research ethics committee (CAPPesq ‒ number 1.888.813/2016), and an informed consent term containing the patient's signature or that of his next-of-kin was previously obtained. This research was supported by *Fundação de Amparo à Pesquisa do Estado de São Paulo* (Process n° 2016/18,394–3), but none of the authors received additional funding.

All transplant candidates were screened for participation in the study. Patients undergoing BDDLT with whole grafts from chronic hepatopathy or acute liver failure were included. Exclusion criteria were refusal to sign the informed consent form before the surgery, partial closure of the abdominal cavity with skin suture, the need for peritoneostomy, simultaneous kidney and liver transplant, contraindications for Indocyanine Green (ICG) dye usage, length of stay at Intensive Care Unit (ICU) up to 48 hours, inability to install the Intra-Abdominal Pressure (IAP) monitoring device (AbViser® system) before skin incision and accidental dislodgement.

### Study design

Preoperative demographic characteristics, indication for liver transplantation, Child-Pugh score, previous complications of cirrhosis, and the Model of End-stage Liver Disease were recorded after obtaining the patient's informed consent.

Once the patient was anesthetized and vascular catheters were placed, a three-way indwelling urinary catheter was inserted under aseptic conditions, with one of the lines connected to a closed measurement system dedicated exclusively to the measurement of IAP (AbViser®, ConVaTec, USA). The AbViser® system was connected to a multi-parametric monitor and zeroed at the mid-axillary line. The IAP was measured by infusing 20 mL of sterile NaCl 0.9 % into the vesical bladder through the AbViser® system before the skin incision intraoperatively and every 6 hours from the ICU admission until 72 hours postoperatively, when the indwelling urinary catheter was removed^14^ The APP was calculated by subtracting the IAP from the MAP. All measurements were performed in the supine position at the end of expiration. When necessary, sedatives and neuromuscular blockers were administered. To ensure the accuracy of IAP measurements, all ICU nursing personnel received training on the method. Intra-abdominal hypertension was defined as IAP over 12 mmHg in at least two consecutive measurements, whereas ACS was defined as sustained IAP over 20 mmHg associated with a new organic dysfunction^6^

The variables recorded intraoperatively were the volume of fluids (crystalloids and colloids), the blood products infused, fluid balance, use of vasopressors, diuresis, and graft weight. Surgical and anesthetic duration, cold and warm ischemic times, Donor Risk Index (DRI), and incidence of reperfusion syndrome, defined as a 30 % drop in Mean Arterial Pressure (MAP) following portal vein unclamping, were also recorded^15^ Ascites was identified when the intraperitoneal fluid volume drained at the opening of the peritoneal cavity exceeded 500 mL.

At the ICU admission, simplified Acute Physiological Score version 3 (SAPS 3) and CLIF-SOFA scores were calculated according to standard formulas^16^, ^17^, ^18^ Blood sampling and laboratory exams were performed according to the ICU protocol. During the first three postoperative days, the fluid balance, use of vasopressors, and the need for mechanical ventilation were evaluated. Acute kidney injury was evaluated according to AKIN score three during the first postoperative week, and the need for hemodialysis was registered until the seventh postoperative day. Death, the need for retransplant, and reoperation were analyzed during the hospital stay.

Early graft dysfunction was defined according to the criteria proposed by Olthoff et al^19^ Postoperative liver graft function was evaluated using standard laboratory tests. The ICG clearance was additionally employed to assess liver function on the third and seventh postoperative days. Using a non-invasive monitoring system (LiMON®), Plasmatic Disappearance Rate (PDR) immediately after injection and ICG Retention after 15-minutes of injection (ICGR15) were recorded after a bolus infusion of ICG (0.25 mg/kg diluted in 10 mL of saline solution) in the central venous access^20^^,^^21^

### Statistical analysis

Considering the principle of clinical relevance and the availability of the AbViser® system, the authors intended to follow at least 100 consecutive patients after BDDLT for the first seven postoperative days in this prospective cohort study. The data's normal distribution was evaluated using the Kolmogorov-Smirnov test and a histogram-based visual analysis of the distribution. Qualitative and quantitative data were compared between the groups that developed IAH (IAH^+^) or not (IAH^-^) through the Chi-Square test, Fisher test, and Wilcoxon test. The behavior of IAP and APP during the observation period was compared between the groups using two-way repeated measures analysis of variance (ANOVA), followed by a Bonferroni post-hoc test if statistically significant results were found. Friedman analysis of variance and the Wilcoxon test were used to compare non-parametric repeated measures data.

Independent predictors for the development of postoperative IAH were tested using a binary backward likelihood ratio logistic regression, where the dependent variable was the presence of IAH. Preoperative and intraoperative variables with a p-value <0.2 in the univariate analysis or clinical plausibility were entered into the regression model. Variables were removed from the model if the p-value was >0.05 in any regression step. All statistical analyses were performed using IBM SPSS Statistical Package version 26 (Armonk, NY, USA). Quantitative data were reported as mean ± standard deviation or median [interquartile range 25 %–75 %], and qualitative data were reported as count ( %) or as otherwise reported. The p-values were considered significant if lower than 0.05.

## Results

**Fig. 1:**
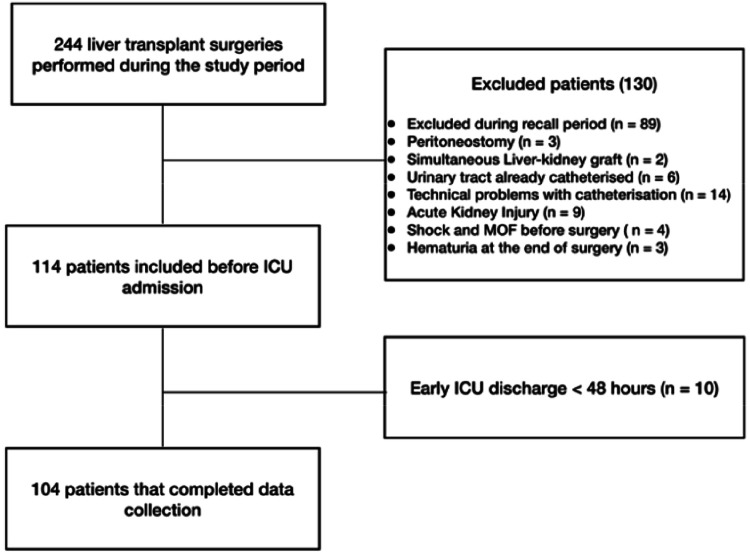
Flowchart showing the patient screening and selection during the study period.

As demonstrated in the flowchart, 244 patients received transplants during the study period. One hundred thirty patients were excluded from the study due to clinical or technical issues, and ten patients were discharged from the ICU in less than 48 hours, preventing the collection of complete data (Fig. 1). From the 104 patients that completed data collection, 15 developed postoperative IAH (14.4 %), and there were no cases of ACS.

### Clinical and epidemiological characteristics of the patients

The characteristics of the patients included in the final analysis are presented in Tables 1 and 2. Most liver transplants were performed in cirrhotic patients (*n* = 95), while 9 were in acute liver failure cases. Of the patients included in the final analysis, 20 were hospitalized before receiving the liver graft due to acute liver failure or Acute-on-Chronic Liver failure (ACL).

Intraoperative data for the entire cohort are presented in Table 2. All patients were operated on using the piggyback technique with a temporary portocaval shunt, with an average surgical time of 440±129 minutes. After anesthesia induction, the mean intra-abdominal pressure was 7 ± 4 mmHg, similar in both groups. The presence of ascites > 500 mL at the opening of the peritoneal cavity was frequent in the IAH^-^ group (47.2 % vs. 6.7 %, *p* = 0.049).

As shown in Table 2, the volume of crystalloid and colloidal solutions infused intraoperatively was comparable between the two groups. However, intraoperative blood product transfusion was more common in the IAH^+^ group, as was the number of patients receiving multiple blood component units. All patients were admitted to the Intensive Care Unit (ICU) using mechanical ventilation and vasopressors. The severity scores of SAPS-3 and CLIF-SOFA were similar in both groups at ICU admission.

### Intra-abdominal hemodynamics, fluid balance, and liver graft function in the first 72-hours after the surgery

**Fig. 2:**
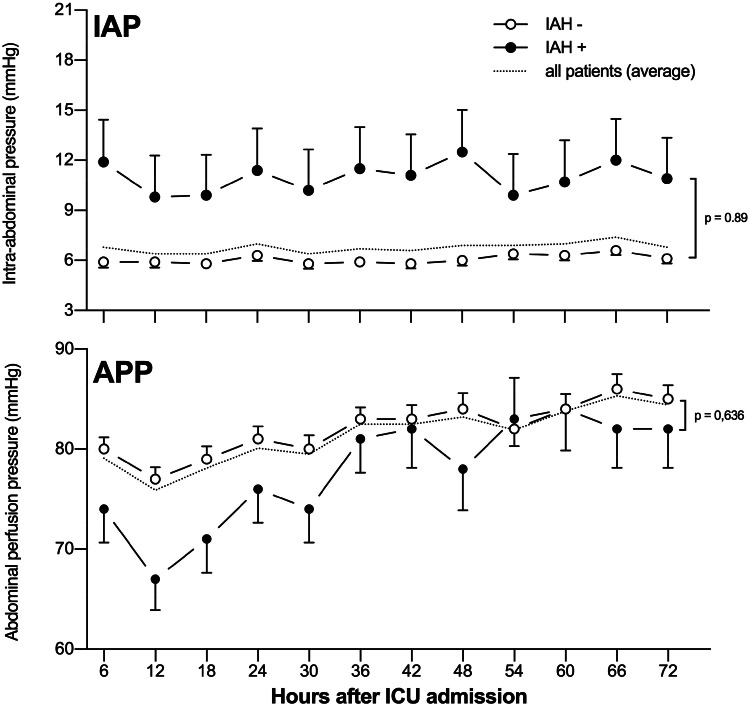
Intra-abdominal pressure (IAP ‒ upper panel) and intra-abdominal perfusion pressure (APP ‒ lower panel) were displayed over the study period. The dotted line represents the average values of all patients in the cohort, black circles represent patients with Intra-Abdominal Hypertension (IAH), and white circles represent patients without IAH. Groups without (IAH^-^) and with (IAH^+^) intra-abdominal hypertension presented as mean ± standard error. The p-value shown refers to the interaction in two-way RM ANOVA.

**Fig. 3:**
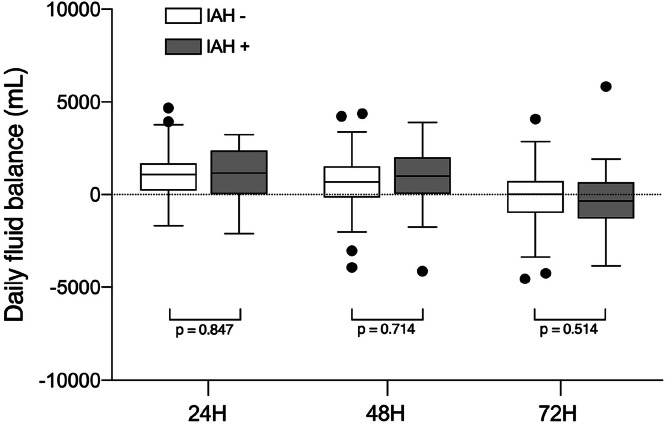
Daily fluid balance in the groups with (gray box-plot) and without (white box-plot) Intra-Abdominal Hypertension (IAH). The central line of the box plot represents the median, and the upper and lower lines represent the 75 % and 25 % interquartile, respectively. Error bars represent interquartile 1.5, and black circles show outliers.

Of the 15 patients who fulfilled the criteria for postoperative IAH, 9 cases (60 %) occurred in the first 24 hours postoperatively, 3 within 48 hours (20 %), and 3 within 72 hours (20 %). All patients with IAH had persistently elevated intra-abdominal pressure throughout the study period, with mean values ranging from 10 to 13 mmHg. In contrast, patients from the IAH^-^ group had pressures ranging from 5 to 6 mmHg (*p* < 0.001), with both groups presenting a constant behavior over time (*p* = 0.89), as shown in the upper panel of Fig. 2. The APP was initially lower in the first 18 postoperative hours in both groups, increasing progressively throughout the study (*p* < 0.001). Patients without IAH had higher mean APP values than those with IAH (*p* < 0.001). However, during the entire observation period, the APP of the group IAH^+^ was within the normal range, consistently over 60 mmHg (Fig. 2). Daily net fluid balance was comparable in both groups in the first 3 days after the surgery, approaching zero on the second and third postoperative days, as shown in Fig. 3.

At days 3 and 7 post-transplant, there was no difference in liver graft function between groups, as measured by ICG kinetics. On the third postoperative day, the median PDR values were (16.5 [12.4‒22.6 %/min] vs. 18.2 [8.3‒29.3 %/min]; *p* = 0.95), and the median ICGR15 values were (8.4 [3.3‒15.9 %] vs. 6.8 [1.7‒28.4 %]; *p* = 0.94] in the IAH- and IAH+ groups, respectively. On the seventh postoperative day, PDR (18.8 [12.9‒24.5 %/min] vs. 17.7 [12.3‒28.6 %/min]; *p* = 0.84) and ICGR15 (6 [2.7‒14.7 %] vs. 7 [1.4‒16.9 %]; *p* = 0.79) exhibited similar behavior in the IAH^-^ and IAH^+^ groups, respectively. The sequential laboratory evaluations of liver transplant function during the first 72 hours revealed no significant differences between the groups.

### Postoperative outcomes and risk factors for IAH

All patients were admitted to the ICU on mechanical ventilation and were receiving vasopressor support. After 48 hours, 60 % of the patients with IAH were still on Mechanical Ventilation (MV) compared to 26.7 % of those without IAH (*p* = 0.01). After 72 hours, 40 % of those with IAH were on MV compared to 13.5 % without IAH (*p* = 0.02).

Seventy patients developed postoperative renal dysfunction (67.3 %), 12 in the group IAH^+^ and 58 in the group IAH^-^ (*p* = 0.355). The proportion of patients with IAH who reached AKIN stage III was 53.3 % (*n* = 8), while those without IAH were 28 % (*n* = 25). Those patients with IAH were more likely to require dialysis at the end of the first postoperative week (31.3 vs. 10.8 %; *p* = 0.047). In the IAH^-^ and IAH^+^ groups, the rates of reoperation (12.4 % vs. 20 %; *p* = 0.42), re-transplantation (10.1 % vs. 13.3 %; *p* = 0.65), and death (14 % vs. 18.2 %; *p* = 0.49) were comparable. The median postoperative ICU and hospital length of stay were 5 (3‒8) days and 14 (9‒25) days in the IAH^-^ group and 5 (4‒10) days and 16 (9‒37) days in the IAH^+^ group, with no statistical difference between groups.

In the multivariable logistic regression analysis, three independent predictors of intra-abdominal hypertension were identified: Body Mass Index (BMI) (OR = 1.185; 95 % CI 1.042–1.348; *p* = 0.010), qualitative ascites (OR = 0.186; 95 % CI 0.042–0.815; *p* = 0.026), and intraoperative blood product transfusion (OR = 1.097; 95 % CI 1.021–1.178; *p* = 0.011). The model showed good overall performance (Nagelkerke R² = 0.301; −2 log likelihood = 66.161) and adequate calibration (Hosmer-Lemeshow χ² = 4.20, *p* = 0.839), with an overall accuracy of 83.3 %, specificity of 96.6 %, and sensitivity of 6.7 %. Table 3 shows these independent risk factors for the development of IAH. Body mass index and the number of blood component units transfused were independently associated with IAH, whereas the presence of ascites at the opening of the peritoneal cavity was protective.

## Discussion

In this prospective cohort, the authors observed that the incidence of IAH was 14.4 %, with the majority of the cases detected within the first 24 hours. No cases of ACS were diagnosed. The mean IAP in the IAH^+^ group was about five mmHg higher than in the IAH^-^ group, and this difference was maintained throughout the observation period. No differences between the groups were observed in liver graft function, need for retransplant, death rate, or postoperative length of stay. However, the need for mechanical ventilation and hemodialysis was higher in the first seven postoperative days in the IAH^+^ group.

It has long been recognized that IAH is associated with worse outcomes in patients undergoing major abdominal surgeries, particularly liver transplantation^5^^,^^22^ In previous studies, the incidence of IAH after OLT has been reported to range from 32 % to 48 %^3^^–^^5^ However, the criteria used to define IAH were variable and arbitrary. Using the definitions of the World Society of the Abdominal Compartment Syndrome (WSACS), the authors observed that the incidence of IAH was 14.4 %, whereas no cases of ACS were observed^6^^,^^23^

Several factors may have contributed to the lower incidence of IAH observed in the present study compared to previous liver transplantation research. The pathophysiological understanding of IAH has evolved significantly over the past two decades, resulting in an improved understanding of its causes^6^^,^^24^ Excessive positive fluid balance is particularly important as it is linked to postoperative organic dysfunctions^25^^,^^26^ and the development of IAH in critically ill patients^4^^,^^27^^,^^28^ It has been shown that avoiding excessive fluid balance decreases the incidence of postoperative organic dysfunctions in surgical patients^12^^,^^25^^,^^26^^,^^29^ The authors routinely adopt an intraoperative infusion strategy guided by pulse pressure variation^30^ along with early initiation of norepinephrine to reduce vascular capacitance, which recruits blood volume from the venous unstressed volume^31^^,^^32^ thereby decreasing intraoperative fluid requirements. Second, the restrictive transfusion strategy using a thromboelastography-guided approach to reduce the number of blood products infused intraoperatively and the decreased transfusion-related inflammatory response might have contributed to this finding^33^^,^^34^ Furthermore, one crucial surgical technical aspect is using a temporary portocaval shunt, routinely performed in this service, which decreases the bleeding during the native liver removal and reduces the intestinal venous stasis and, consequently, the intestinal edema during and after the surgery^35^^,^^36^ Other aspects that may have contributed to a lower incidence of postoperative IAH compared to older series are shorter surgical times, better donor-recipient matching, and optimized graft preservation, which reduce the intensity of the surgery-related systemic inflammation^37^^,^^38^ Finally, the increased awareness about the negative impact of IAH/ACS on postoperative outcomes may unconsciously modify the treatment standards^39^

When evaluating the independent predictors of postoperative IAH, intraoperative ascites volumes greater than 500 mL were a protective factor with an Odds Ratio (OR) of 0.19. In contrast, an increased BMI (OR=1.18) and transfusion of blood products (OR=1.1) were directly associated with an increased risk. As portal hypertension worsens during the course of cirrhosis, the increasing ascites volume promotes a progressive expansion of the intra-abdominal compartment capacity, creating more space to accommodate the distended intra-abdominal viscera and fluids, thereby reducing the risk for IAH. Although a physiological association between the amount of intraoperative ascites and baseline Intra-Abdominal Pressure (IAP) could be expected, the limited number of patients with measurable ascites volumes, did not allow a meaningful correlation analysis. For this reason, ascites was analyzed as a categorical variable (> 500 mL vs. ≤ 500 mL), which proved to be independently associated with postoperative intra-abdominal hypertension.

On the other hand, patients without ascites and with normal abdominal cavity compliance, such as those with acute liver failure, may be at a higher risk for developing IAH^40^ The multivariate model also showed that patients with higher BMI are at a greater risk for developing IAH, corroborating previous publications in diverse populations such as those undergoing abdominal surgery and general ICU patients^1^^,^^41^^,^^42^ A distinct association between transfusion and IAH was observed, which might be explained by the volume of blood products infused and transfusion-related systemic inflammation that may worsen tissue edema^43^

Even though the average IAP in the IAH^+^ group ranged from 10 to 13 mmHg, which according to the WSACS definitions^6^ is considered mild IAH, it was associated with an increased need for postoperative mechanical ventilation and renal replacement therapy. Although the present study was not designed to identify causality, it supports the hypothesis that even slight elevations in IAP, resulting in slight reductions in APP, may play an important role in developing postoperative organ failure following liver transplantation. Interestingly, the graft function assessed by the ICG clearance measure did not differ between IAH^+^ and IAH^-^ groups. The impact of slight elevations in IAP, albeit possibly implicated in other organic dysfunctions, might not be enough to hamper hepatic perfusion and intensify ischemia-reperfusion injury in the liver graft.

This study has several limitations: 1) It reflects the experience of a single center specialized in the care of cirrhotic and liver transplant patients with a dedicated ICU; 2) Due to the observational nature of this study, the authors were unable to evaluate some of these practices, such as the temporary portocaval shunt or the restrictive intraoperative fluid strategy. 3) A worldwide recall of the ABVISER® dispositive occurred during the study, resulting in the study's discontinuation for nine months. However, the authors do not believe there was a selection bias since the patients were included consecutively throughout the study. 4) Finally, three patients in whom a peritoneostomy was required due to excessive bleeding were excluded from the study because the IAP could not be accurately measured due to the impossibility of achieving fascial closure. Since the abdominal closure was performed 48h–72 hours after the transplant, and the patients never experienced IAH, their removal of the sample does not alter the results.

In conclusion, the authors observed that the incidence of IAH was low and mild (grade I WSACS definitions), with no cases of ACS. However, even low-grade IAH was significantly associated with a higher postoperative need for mechanical ventilation and renal replacement therapy, while liver graft function patterns were similar between groups (Table 1).

**Table 1:** Clinical and demographical characteristics of the patients undergoing liver transplantation included in the study.

	**All patients** **(*n* = 104)**	**IAH^-^** **(*n* = 89)**	**IAH^+^** **(*n* = 15)**	**p-value**
**Male Gender, n (%)**	63	53 (59.6)	10 (66.7)	0.777
**Age (years)**	50.7 ± 13.4	50.9 ± 13.6	50.1 ± 12.2	0.697
**Weight (Kg)**	74.5 ± 15.1	73.3 ± 14.4	81.7 ± 17.7	0.077
**Height (cm)**	167.2 ± 8.5	167.6 ± 8.5	165.1 ± 8.8	0.23
**BMI (Kg/m^2^)**	26.6 ± 4.7	26.0 ± 4.5	29.8 ± 4.9	0.007
**Transplant indication, n (%)**				
HCV	33 (27.2)	29 (28.7)	4 (20)	0.77
Álcool	30 (24.7)	24 (23.7)	6 (30)	0.359
NASH	16 (13.2)	15 (14.8)	1 (5)	0.457
Criptogenic	12 (9.9)	11 (10.8)	1 (5)	1
ALF	9 (7.4)	6 (5.9)	3 (15)	0.12
Autoimmune	7 (5.7)	6 (5.9)	1 (5)	1
HBV	2 (1.7)	1 (1)	1 (5)	0.269
Others	12 (9.9)	9 (8.9)	3 (15)	0.12
**Cirrhosis complications, n (%)**				
Hepatic encephaloty	46 (44.2)	40 (44.9)	6 (40)	0.785
SBP	25 (24)	24 (27)	1 (6.7)	0.111
Hepato-Renal Syndrome	17 (16.3)	15 (16.9)	2 (13.3)	1
**CHILD-PUGH Score, n (%)**				0.213
A	25(24.0)	22 (27.2)	3 (27.3)	
B	38(36.5)	32 (39.5)	6 (54.5)	
C	29(27.9)	27 (33.3)	2 (18.2)	
No data available	12(11.5)	8 (9)	4 (26.7)	
**MELD score, n (%)**				0.346
< 20	65 (62.5)	58 (65.2)	7 (46.7)	
20‒29	17 (16.3)	15 (16.9)	2 (13.3)	
30‒39	18 (17.3)	13 (14.6)	5 (33.3)	
≥ 40	4 (3.8)	3 (3.4)	1 (6.7)	
**Pre-op. creatinine (mg/dL)**	0.96 [0.74‒0.41]	0.94 [0.75‒1.28]	1.15 [0.58‒3.27]	0.347

IAH, Intra-Abdominal Hypertension; BMI, Body Mass Index; HCV, Hepatitis C Virus; NASH, Non-Alcoholic Steatohepatitis; ALF, Acute Liver Failure; HBV, Hepatitis B Virus; SBP, Spontaneous Bacterial Peritonitis; MELD, The Model of End-stage Liver Disease.

* Percentages may not total 100 % due to rounding.

**Table 2:** Intra-operative data and admission ICU severity scores.

	**All Patients** **(*n* = 104)**	**IAH^-^** **(*n* = 89)**	**IAH^+^** **(*n* = 15)**	**p-value**
**IAP before incision (mmHg)**	7 ± 4	7 ± 4	8 ± 5	0.334
**Intraoperative fluid balance (L)**	3 [1.5‒3.8]	3 [0.9‒3.8]	3.2 [2.4‒3.9]	0.441
**Intraoperative ascitis >500 mL, n (%)**	45(43.3)	42 (47.2)	3 (6.7)	0.049
**Anesthesia duration (min)**	586 ± 133	559 ± 89	548 ± 86	0.893
**Surgery duration (min)**	440 ± 129	413 ± 84	407 ± 96	0.842
**Cold ischemia duration (min)**	446 ± 147	472 ± 116	496 ± 146	0.929
**Warm ischemia duration (min)**	31 ± 4	31 ± 5	30 ± 3	0.658
**Graft weight (g)**	1322 ± 358	1328±361	1290 ± 354	0.711
**Donor Risk Index**	1556 ± 343	1561±358	1527 ± 238	0.739
**Intra-op. crystaloid volume (mL)**	4354 ± 1563	4411±1597	4017 ± 1343	0.368
**Intra-op. 20% albumin solution (mL)**	302 ± 157	297 ± 153	332 ± 186	0.443
**Pacients transfused, n (%)**	74 (71.2)	59 (66.3)	15 (100)	0.008
**Blood products used, n (%)**				
PRBC	70 (68)	56 (63)	14 (93.3)	0.02
Fresh frozen plasma	32 (30.8)	24 (27)	8 (53.3)	0.055
Platelet apheresis	42 (40.4)	34 (38.2)	8 (53.3)	0.269
Crioprecipitate	17 (16.3)	10 (11.2)	7 (46.7)	0.001
**Politrasfusion ≥ 4 BPU, n (%)**	54 (51.9)	42 (47.2)	12 (80)	0.019
**Reperfusion syndrome, n (%)**	41 (39.4)	35 (39.3)	6 (40)	0.961
**Noradrenaline infusion, n (%)**	104 (100)	89 (100)	15 (100)	‒
**SAPS3 score (ICU admission)**	62 ± 9	62 ± 9	63 ± 5	0.627
**CLIF-C OF score (ICU admission)**	12 ± 3	12 ± 3	13 ± 3	0.087

IAP, Intra-Abdominal Pressure; PRBC, Packed Red Blood Cells unit; BPU, Blood Product Unit; SAPS3, Simplified Acute Physiological Score ‒ version 3; CLIF-C OF, Chronic Liver Failure Consortium Organic Failure score.

**Table 3:** Preoperative and intraoperative independent risk factors for the development of intra-abdominal hypertension.

	**OR**	**95% CI**	**p-value**
**BMI (Kg/m^2^)**	1.18	(1.04 ‒ 1.35)	0.01
**Intraoperative ascitis > 500 mL**	0.19	(0.04 ‒ 0.82)	0.02
**Blood products units (n)**	1.10	(1.02 ‒ 1.18)	0.01

BMI, Body Mass Index.

* BMI and blood product transfusion were modeled as continuous variables. ORs therefore represent the change in odds per 1 kg/m² increase in BMI and per 1 unit of blood product transfused.

## Data availability

The datasets generated and/or analyzed during the current study are available from the corresponding author upon reasonable request.

## Declaration of competing interest

The authors declare no conflicts of interest.
